# "The Hospital" Medical Book Supplement—No. XXVII

**Published:** 1910-03-19

**Authors:** 


### "THE HOSPITAL" MEDICAL BOOK SUPPLEMENT—No. XXVII.

CONTENTS.

| | Page |
|---|---|
| **Special Article—** | |
| The Story of the Hospitals ... ... ... ... ... ... | 723 |
| **Medicine—** | |
| The Conquest of Consumption ... ... ... ... ... ... | 724 |
| Epilepsia ... ... ... ... ... ... ... ... ... ... | 724 |
| A Plea for the Home Treatment and Prevention of Scarlet Fever ... ... ... ... ... ... ... ... ... | 724 |
| **Surgery—** | |
| A Synopsis of Surgery ... ... ... ... ... ... ... ... | 725 |
| Rhinology: A Textbook of Diseases of the Nose and the Nasal Accessory Sinuses ... ... ... ... ... ... ... ... | 725 |
| Syphilis ... ... ... ... ... ... ... ... ... ... | 725 |
| **Miscellaneous—** | |
| Infancy ... ... ... ... ... ... ... ... ... ... | 726 |
| Alcohol: Its Effects upon the Organs of the Body ... ... | 726 |
| First Aid to the Injured and Sick: An Advanced Ambulance Handbook ... ... ... ... ... ... ... ... ... | 726 |

### SPECIAL ARTICLE.

### THE STORY OF THE HOSPITALS.

A History of the London Hospital. By E. W. Morris. (London: Edward Arnold. 1910. 6s.)

We have frequently urged the importance for every voluntary hospital with a hundred years' work or more behind it to have its history written and published. Mr. Morris has had a fascinating subject in the London Hospital at Whitechapel, which is the largest British voluntary hospital. Its beginnings, its early struggles, the unhygienic conditions under which the work had to be done, the extraordinary ignorance which those conditions revealed on the part of hospital authorities in the days in which they prevailed, the many difficulties which had to be faced, the horrors of the cholera years with the morning call of Pickford's vans for the dead, and a multitude of other incidents make this book full of interest. Mr. Morris writes pleasantly, tersely, and well. He evidently has imbued himself with the spirit of the place, and the result is a book from which we do not intend to make extracts in detail, because our readers and all who are interested in hospitals should make a point of reading it. It would not be difficult to fill several columns with extracts which have a permanent interest, but such a course would be an injustice to the author, who is entitled to the reward of a large circulation for a book which must have cost him a great deal of labour and involved the perusal of many documents and much written matter not easy to study continuously and deal with.

The book bears evidence of the exercise of the judicious spirit in handling many points where individuals are concerned. It records with faithfulness transactions which have a much wider importance than even their connection with this great hospital would undoubtedly give them. One such notable instance is the account given on p. 226 of the immediate cause which led to the rebuilding of the London Hospital in 1897 and subsequent years. There it is stated that this scheme of rebuilding originated in a correspondence between the King's (then the Prince of Wales's) Hospital Fund and the House Committee of the London Hospital. The Fund stated in a letter to the Committee dated December 29, 1897, that if the hospital would spend £100,000 of its capital and place it to the credit of the Building Fund, the King's Fund would pay to the hospital an annual subscription of £5,000 a year. The London Hospital has now been thoroughly reconstructed and rebuilt, and the King's Fund has continued this annual subscription of £5,000 a year ever since. If the work had to be done again there is little doubt that it would be undertaken in sections from a perfected plan first settled for the rebuilding of the whole institution in all its departments. The task of rebuilding this hospital and providing it with 1,000 beds was, however, large enough in 1897 to appal anyone who had a hand in it, especially when the committee, with a laudable desire to help the sick poor of East London, resolved that the building operations should not diminish the accommodation for in-patients by a single bed from start to finish.

Mr. Morris pays just tribute to Mr. Sydney Holland, who took up the responsibility of the chairmanship at a time when the hospital was passing through its most strenuous years, when everything was pressing and at every meeting of the committee some new development had to be considered. Money was sadly wanted. Mr. Sydney Holland threw himself into the work, and by the interest he displayed in it he fired a number of people with his own enthusiasm for the work which had to be undertaken. He so overcame all difficulties, gave the London Hospital an up-to-date building, and made things possible which before his advent no one could be got to face. Mr. Morris declares that Mr. Holland has been Chairman of the hospital since 1896, and that at the end of 14 years he works as hard for the hospital as he did at the commencement. It is no small thing to have accepted the chairmanship at a stage in the hospital's history "which made the most sanguine despair. Never was money so wanted since the hospital began. There was none. Even ordinary upkeep could not be met." As Mr. Morris records, that time was the darkness before the dawn, for under Mr. Holland's chairmanship every department has been brought up to date, new departments have been added, and the whole institution, though it needs an increased revenue of £35,000 a year, is to-day one of the most remarkable and well supported hospitals in the world. Mr. Morris also pays a high tribute to Miss Luckes, the present matron, who introduced proper and systematic training for nurses at this hospital in 1880, and induced members of the medical and surgical staff to teach the nurses too. She it was who instituted Tredegar House, the preliminary training school for nurses, and Mr. Morris declares of Miss Luckes that "no living woman has done more for the betterment of the conditions under which nurses work, the shortening of their hours, the lengthening of their holidays, and the improvement of the domestic arrangements for their comfort."

Mr. Morris concludes a most interesting book by emphasising the value of the hospital spirit, a spirit which is common to every British hospital which is administered with zeal and efficiency by people who believe that personal service in the cause of the sick is a privilege which all thinking men and women owe to their individuality as an offering of praise to the great Giver of all good, for the blessings of health. The book is well printed, but we hope that in future editions the index may be amplified so that all the subjects dealt with and individuals referred to may be included. A book recording the history of a great hospital cannot be complete unless the index errs, if it errs at all, on the side of fulness, because such books, if they are well done, must constitute a most helpful portion of the reference branch of every subject library. We congratulate Mr. Morris upon the ability he has displayed. We commend this book to everybody, high and low, connected with a hospital who has any appreciation of the spirit which makes association with such a place full of charm and benefit to some of the best members of the race.

### MEDICINE.

The Conquest of Consumption. By Arthur Latham, M.D., and C. H. Garland. (London: T. Fisher Unwin. 1910. Price 4s. 6d. net.)

Dr. Latham and Mr. Garland quite correctly describe their joint production as an Economic Study, for that is what it is, and a very striking one as well. To certain points connected with this book reference has already been made in these pages (see The Hospital, February 19, 1910, p. 595, and there is no need to repeat what was there set forth. Nor need we consider in detail the economic problems which pulmonary consumption in these islands has produced, for they are exhaustively discussed by the authors. The work is one with which medical men should be familiar—it is easily read in an hour—for it touches on many aspects of tuberculosis which are the least familiar to the profession, and should provide much food for thought. For economists it can be even more confidently recommended, and we sincerely trust it will have a wide circulation among them.

Epilepsia. (London: Williams and Norgate. Price 18s. per annum.)

This is an international quarterly review devoted exclusively to epilepsy and its allied nervous maladies from their pathological, therapeutical, social, and medico-legal points of view. It is under the patronage of such well-known authorities as Bechterew, Binswanger, Hughlings Jackson, Luciani, Obersteiner, and Raymond. It is well printed upon light but good paper in large type, and the green paper cover lettered down the back is sufficiently good to permit of the volumes being kept upon one's shelves without binding annually. The degree to which specialisation in the different departments in medicine is being carried nowadays is brought home to one strikingly when one finds that special periodical publications are beginning to appear upon such comparatively small sub-divisions as "Heart," which we reviewed recently, and "Epilepsy," which we are now reviewing. Nevertheless, so numerous are the papers that are published nowadays throughout the world that it has become necessary to sift them at comparatively short intervals, and publish periodically the gist of those which are important. A work of this kind is likely to appeal chiefly to specialists however, rather than to the general practitioner, especially as several different languages are employed, some of the papers appearing in German, others in French, and only a few in English.

The numbers before us, besides summarising the current literature upon epilepsy, contain several original articles, and each of these terminates with a concise summary in some other language of the main points dealt with in it. Thus the article by Raymond and Serieux upon "La Responsabilité et la Condition Sociale des Epileptiques" is summarised in German; Redlich's "Bemerkungen zur Alkohol-Epilepsie" in English; Muskens' "Prodromal Motor Sensory and Other Symptoms and their Clinical Significance" in German, and so on. In the second number Binswanger writes upon "Aufgaben und Ziele der Epilepsie-Forschung"; McDougall gives an account of the David Lewis Manchester Epileptic Colony; Donath discusses "Der Wert des Chlorcalciums in der Behandlung der Epilepsie"; Muskens deals with "Regional and Myoclonic Convulsions"; and Apelt summarises recent literature upon hystero-epilepsy. In the third number Marie contributes an article upon "Ligue Internationale contre Epilepsie"; Hebold writes "Ueber Epileptiker-anstalten"; and Muskens upon "Neuere Ergebnisse des Segmentalen Sensibilitätsuntersuchungen." It remains to be seen whether the excellence of the original contributions can be maintained in future years; one fears that as time goes on there will be little fresh left to say, in which case the value of the publication will diminish. Time alone will show whether this is to be so or not, and meanwhile we commend this quarterly publication to those who are specially interested in epilepsy.

A Plea for the Home Treatment and Prevention of Scarlet Fever. By Robert Milne, M.D. (London: James Nisbet and Co., 1910. 80 pages. Price 2s.)

The author of this brochure believes that the contagiousness of scarlet fever is easily and certainly reduced to vanishing point by the adoption of a regular routine of antiseptic prophylaxis. For the first four days he has his patients rubbed from the crown of the head to the soles of the feet twice a day with pure eucalyptus oil, and then once a day until the tenth day of the disease. The tonsils are swabbed with 1 in 10 carbolic oil every two hours for the first twenty-four hours. He holds that if these measures are properly carried out there is no necessity for the isolation of the sufferer after the first ten days, and no necessity for his removal to a fever hospital. Bedding, linen, etc., used before the inauguration of the treatment needs sterilisation; but that subsequently used does not. Books, toys, letters, clothes, and other articles which have frequently been blamed as vehicles of infection, are also harmless if the patient is thus disinfected; every part of the face is to be treated, in fact every part of the entire body. The peeling stage is said to be shortened, and the severity of the disease mitigated. These opinions the author has formed as the result of very prolonged experience in institutions and in private practice, and revolutionary as they may appear, they cannot lightly be set aside without careful investigation. They are by no means new, for the author has already formulated them in the *British Medical Journal* and elsewhere. There is no doubt possible of Dr. Milne's sincerity; his somewhat obscure sentences breathe that, if they do nothing else. Nor is the apparent audaciousness of his scheme any cogent argument for rejecting it. The method must stand or fall by its merits, and the issue of this book is welcome as affording the profession an opportunity of hearing his claims and putting them to practical tests. Any medical officer to a large institution of susceptible children should certainly give the plan a trial next time an epidemic breaks out.

### SURGERY.

A Synopsis of Surgery. By E. W. H. Groves, M.S., M.D., F.R.C.S. Second Edition; Revised and Illustrated. (Bristol: J. Wright and Sons., Ltd. 1910. Pp. 579. Price 9s. 6d. net.)

The amazing growth of modern surgery is aptly illustrated by the fact that this bare outline of examination notes for students comprises actually 579 pages. Yet the synopsis of surgery as here presented is a condensation carried to a degree which restricts its usefulness entirely to the needs of those who are just taking a last hurried revision before sitting for an examination in surgery. That it has found favour with medical students is evident enough, for the new edition has been required within eighteen months of the first publication of the original. This popularity is, we think, deserved; but we hope that the general practitioners to whom the author addresses himself in the preface have not aided very materially in exhausting the first edition, for "tabloid" surgery of this nature, however convenient to the examinee, is not of much practical utility to the qualified man. Sections have been added to this edition on asepsis and antisepsis, shock, anæsthetics, and diseases of the colon. In the chapter on anæsthetics is a good instance of the pitfalls which await the author of student guides of this sort. Rightly dogmatic, and rightly cautious in describing chloroform anæsthesia to unqualified men, the writer of this chapter (Dr. John Freeman) actually recommends that if the pupil of a patient inhaling chloroform be dilated and unresponsive to light, the proper treatment is to suspend administration and begin artificial respiration at once without waiting for signs of respiratory failure. We venture to doubt whether Dr. Freeman practises this himself, and we cannot anyhow see the use of artificial respiration for a patient whose breathing shows no signs of inefficiency. The fact of the matter is that the subject of anæsthesia is one that cannot profitably be dealt with in the brief summary which is all that considerations of space permit in a work of this sort. The other new sections are all good. A strong feature of the synopsis is the excellent index, and the very great attention which has been paid to classification, by judicious selections of type, indentation, and arrangement, this last virtue is displayed to the utmost advantage.

Rhinology: A Textbook of Diseases of the Nose and the Nasal Accessory Sinuses. By Patrick Watson Williams, M.B. (London: Longmans, Green and Co. 1910. Pp. 273. Price 15s. net, with stereoscope; 12s. 6d. without.)

The well-known textbook on the nose and throat, of which this author has published four editions, having grown to unwieldy dimensions, the decision to separate the two specialties by devoting a separate monograph to each is indubitably a sound one. In rhinology especially, the advances made in quite recent years have been remarkable, and the author points out that the present work is of twice the dimensions to which the corresponding section of the old textbook had attained in the last edition. The companion volume on Laryngology is promised shortly, and may be expected to prove an even more extensive treatise than that now under consideration.

The most noticeable feature of Dr. Watson Williams' new work is the quantity and quality of the illustrations, of which there are one hundred and forty-six in the text as well as forty-seven plates, the majority of which are stereoscopic. This lavish provision is due to the author's conviction—one with which we entirely concur—that the anatomy of the nose and of its accessory sinuses is a subject so intricate and so important that the whole science of operative rhinology hinges upon it. The variations in the dispositions of these organs in normal individuals are probably greater than those of any other section of the human body, and no one who neglects their most attentive study can hope to escape disaster if he attempts to treat cases of sinus disease. The stereoscopic plates provided go far towards laying the necessary foundations of this minute anatomical knowledge, but they have the disadvantage of requiring a particularly heavy paper, which makes the volume much too heavy for comfortable reading. As far as the text goes, practitioners will find a good deal about operations and conditions which most of them are quite content to leave to specialists; but on the other hand there is no neglect of those common complaints which frequently fall to their lot to treat. It is interesting, for instance, to read the author's reasons for reversing the usual custom of removing tonsils before tackling adenoids. The consideration of the difficulties, dangers, failures, and complications of this operation is thorough, but a little more about after-treatment would not have been amiss. We are glad to notice the very common-sense attitude taken up on the subject of asthma and its connection with nasal affections: Dr. Williams condemns the exaggerated statements of certain enthusiasts, according to whom any and every case of asthma can be certainly cured by a cauterisation of appropriate spots on the septum nasi. At the same time he appreciates fully the benefit to be derived from the removal of nasal polypi when they exist, or the treatment of septal deformities which cause contact of the overlying mucosa with that of the turbinals. He well remarks that the true ætiology of the association is more likely to be that polypi and asthma are both the result of some prime cause (possibly in some cases ethmoid suppuration) than that either sets up the other. The companion volume on Laryngology will be awaited with an interest very considerably whetted by the excellence of this practically new textbook.

Syphilis. By Sir Jonathan Hutchinson, F.R.S., LL.D., F.R.C.S. New Edition. (Cassell and Co., Ltd. 10s. 6d. net.)

The first edition of this well-known and deservedly popular manual was published more than twenty years ago, and though many reprints have appeared from time to time, no revised edition has been issued since 1887. During these twenty odd years our theories concerning the disease and its treatment have been largely modified, much new information has been published, and the mass of literature dealing with this subject has grown to such an extent that it is a physical impossibility, even for one who specialises in syphilology, to be conversant with everything that has been written and said during these two decades. It is, therefore, an indisputable mark of excellence for a manual such as this that the new edition, which lies before us, shows no broad general modification of opinion such as might reasonably have been expected by one who was ignorant of the careful work which Sir Jonathan Hutchinson gave us in 1887. In one sense it is, of course, inevitable that the author should have re-written some parts of the work in order to keep abreast of modern teaching, but it is a remarkable fact that the main portion of the book remains as it was in the first edition—in other words, that the clinical, as differentiated from the pathological, conception of syphilis has remained so constant. That this is a fact, so far as this book is concerned, is largely due to the painstaking care, the wide knowledge, and the excellent judgment displayed by the author in the first edition. The new edition, like the old, is eminently suitable for the general practitioner. Here he will find, condensed in a readable fashion (for Sir Jonathan Hutchinson has the gift of presenting his facts with a terse clearness and vigour which should be an example to other surgeons who write textbooks), the gist of our knowledge of syphilis, its ætiology, pathology, clinical course, variations, and, what is specially important, its treatment. Moreover he will not be browbeaten by pages of theoretical matter, which, however interesting to experts, are usually skipped by the reader who takes up such a book for the purpose of getting practical guidance. The niceties of serum diagnosis, for instance, are briefly dealt with in the appendix, where the reader who wishes for fuller information on these and other abstruse points is referred to the appropriate literature. The discovery of the Spirochæta, as Sir Jonathan justly observes, "had been so confidently foreseen, and had become by inference so definitely interwoven with the texture of our creed that now that it has been actually made it brings us but little help." The preface, in fact, sums up the modern position fairly and clearly, and should be read by all those who read the text of the book, while in the appendix various themes are touched upon which are all of interest, and many of which possess great practical value. The clinical identity of Bazin's disease with certain cases of multiple ulceration of the legs, described in the first edition, is admitted by Sir Jonathan in this. The chapter on nervous disorders is thoroughly revised, and one of the best in the book. The sections dealing with treatment are equally excellent, and the author's memoranda on the use of iodides offer many valuable practical hints. We are glad to note that the author does not favour the use of atoxyl and soamin, and to note also, that he holds that Fowler's solution in small doses is a valuable adjuvant in some cases. The arsenic treatment has been very popular of late years, and practitioners are apt to forget that older drugs which they possess in their surgery are probably as valuable. It must be remembered that Sir Jonathan Hutchinson takes a view as regards syphilis, which is usually regarded as being too optimistic. In this, however, he will be supported by the experience of every general practitioner. The medical student and the hospital surgeon see, perhaps, the worse types of the disease and its protean variations. In private practice, however, the dark side of the shield is not so prominently displayed, and the practitioner is always inclined to take a more favourable view. The reading of this book will strengthen that impression. With the many illustrative cases given, with its really useful illustrations, its clearly laid down lines of treatment, and its equally clear descriptions, it is a book that will prove of real help and value to everyone who is interested in the disease, and it can be confidently recommended to such practitioners as are not already familiar with it. It is one of the cheapest books, considering the amount of information it contains, to be bought from medical publishers.

### MISCELLANEOUS.

Infancy. Edited by T. N. Kelynack, M.D. (London: Robert Culley. 1910.)

This publication is the first of a series of National Health Manuals, intended to afford concise and up-to-date scientific presentation of the principles and practices which guide and govern the establishment and maintenance of personal, domestic, and national health. This somewhat magniloquent pronouncement is quoted from Dr. Kelynack's preface, from which it is also clear that the volumes are intended for the lay public. As for the present work, there can be no doubt that a highly intelligent parent of either sex may profit very greatly by a careful study of it: but a high grade of intelligence is, according to Carlyle, not very frequently encountered among the population, and it seems pretty certain that the majority will be a good deal mystified. Of course a dogmatic tone is unavoidable when dealing popularly with an abstruse science; for that very reason immense care should be taken to include no statement to which exception can be taken, and we have found several of them. The editor, too, has not been quite careful enough in restricting the use of technical phraseology by his contributors. Apart from these defects, the volume is a most instructive synopsis of infant problems from every point of view. We wish the promoters of the series every possible measure of success in their endeavours on behalf of the national health, and that this present manual may help towards the attainment of their ideals.

Alcohol: Its Effects upon the Organs of the Body. By H. A. Lediard, M.D., F.R.C.S. (Carlisle: Chas. Thurnam and Sons. English sheet. Price not stated.)

This is a reprint of a public lecture delivered by Dr. Lediard against alcohol. It deals with the various aspects of the temperance question in a common-sense, plain-spoken manner, avoiding the unrestrained enthusiasm—to use a mild term—of the extremist. As we are principally concerned with the medical aspect of the question, we naturally turn to that part of the lecture where Dr. Lediard explains his views on alcohol as a drug. His argument here is, in our opinion, unconvincing, because he takes no account of the fact that alcohol is a very valuable drug used as a drug. In cases of pneumonia it is an undoubted fact that alcohol is one of the best—some practitioners would go so far as to say the best—last-stand stimulant that we know of; strychnine, camphor, and caffeine injections may, and undoubtedly do, supply its place on occasions, but those who have had experience in such cases still agree that alcohol is a most valuable drug in these conditions, and that it would be folly to scratch it off the drug list. We have read this little book, which is printed in a model manner, with much interest and pleasure, not only because Dr. Lediard presents his facts in an attractive form, but because throughout the work he maintains a calm and judicial mind which greatly enhances the value of his conclusions.

First Aid to the Injured and Sick: an Advanced Ambulance Handbook. By F. J. Warwick, M.B., and A. C. Tunstall, M.D. Sixth Edition. (Bristol: J. Wright and Co. 1910. Price: Leather, 2s. 6d. net, paper, 1s. net.)

The popularity of this well-known handbook is so widespread that in drawing attention to the sixth edition we need scarcely say more than that it has been thoroughly revised, and the chapter on poisons rewritten. An improvement is also to be seen in the matter of illustrations, which are much more helpful now than were some of those in the original edition, and also much more numerous. An innovation is the introduction of six coloured illustrations. The incorporation of much of the Stretcher and Wagon Drill from the "Royal Army Medical Corps Training" of 1908, is also an advantage which medical officers of Territorial Units who lecture to their men will not be slow to appreciate.

